# Leadership During a Pandemic: A Lexical Analysis

**DOI:** 10.3389/fpubh.2022.783337

**Published:** 2022-04-25

**Authors:** Ann Dadich, Abby Mellick Lopes

**Affiliations:** ^1^School of Business, Western Sydney University, Parramatta, NSW, Australia; ^2^School of Design, Faculty of Design, Architecture and Building, University of Technology Sydney, Ultimo, NSW, Australia

**Keywords:** leadership, COVID-19, health service management, Leximancer, review

## Abstract

To manage pandemics, like COVID-19, leadership can enable health services to weather the storm. Yet there is limited clarity on how leadership manifested and was discussed in the literature during COVID-19. This can have considerable public health implications given the importance of leadership in the health sector. This article addresses this missed opportunity by examining the literature on leadership during a pandemic. Following a systematic search of nine academic databases in May 2021, 1,747 publications were screened. Following this, a lexical analysis of the results section was conducted, sourced from a corpus of publications across myriad journals. The results found a prevalence of references to “leader” as a sole actor, risking the perpetuation of a view that critical decisions emanate from a singular source. Moreover, “leadership” was a concept disconnected from the fray of frontline workers, patients, and teams. This suggests a strong need for more diverse vocabularies and conceptions that reflect the “messiness” of leadership as it takes shape in relation to the challenges and uncertainties of COVID-19. There is a considerable opportunity to advance scholarship on leadership via further empirical studies that help to clarify different approaches to lead teams and organizations during a pandemic.

## Introduction

When the World Health Organization (WHO) declared a public health emergency of global concern on March 11, 2020 (1), there were few precedents for the multiple social, economic, and institutional impacts that this pandemic would generate. The need for capable, resilient leaders with strong adaptive capacity would appear to have never been more important. This is particularly the case in the health sector, where the challenges and uncertainty surrounding COVID-19 are magnified (2).

Despite the importance of leadership in the health sector (3–5), there is limited clarity on how leadership manifested and was discussed in the literature during COVID-19. This represents a missed opportunity to learn from recent experiences, particular given the likelihood of future pandemics (6–8). To address this gap, this article examines leadership research pertaining to COVID-19.

Through a lexical analysis of 36 publications, this article offers a snapshot of how leadership, as a concept and practice, was characterized. While the results reveal pathways within the discourse where leadership was demonstrated, experienced, or longed for during the global crisis, they also reveal fertile ground for future research.

## Methods

A search strategy was deployed in nine academic databases in May 2021 to identify all publications on leadership during COVID-19. Given their relevance and comprehensiveness, the following academic databases were included: APA PsycArticles; APA PsycInfo; Business Source Complete; CINAHL Plus with Full Text; Health Business Elite; Health Source: Nursing/Academic Edition; Medline; Psychology and Behavioral Sciences Collection; and SocINDEX with Full Text. The search strategy encompassed leade^*^ and terms that denote COVID-19 (i.e., coronavirus, COVID-19, pandemic, or SARS-CoV-2) within the title and/or abstract of the publication to optimize the relevance of each publication. Alternative terms that potentially denote leadership were purposely absent from the search strategy to optimize coherence—for instance, although the terms, management, administration, supervision, and authority, might be relevant, they are not synonymous with leadership—as such, they did not form part of the search strategy to optimize comparability among the publications that were identified. Publications were included in this review if they: pertained to COVID-19; were published in English; represented an empirical study (regardless of whether it involved the analysis of primary or secondary data), rather than a literature review (including systematic reviews and meta-analyses), a conceptual study, a discursive article, a study protocol, an editorial, a commentary, or a book review; were authored; and represented a refereed journal publication. This study purposely focused on empirical studies, irrespective of research design, to clarify patterns in academic discourse on how leadership was portrayed – as such, gray literature and policies were not included in this lexical analysis.

**Figure 1:**
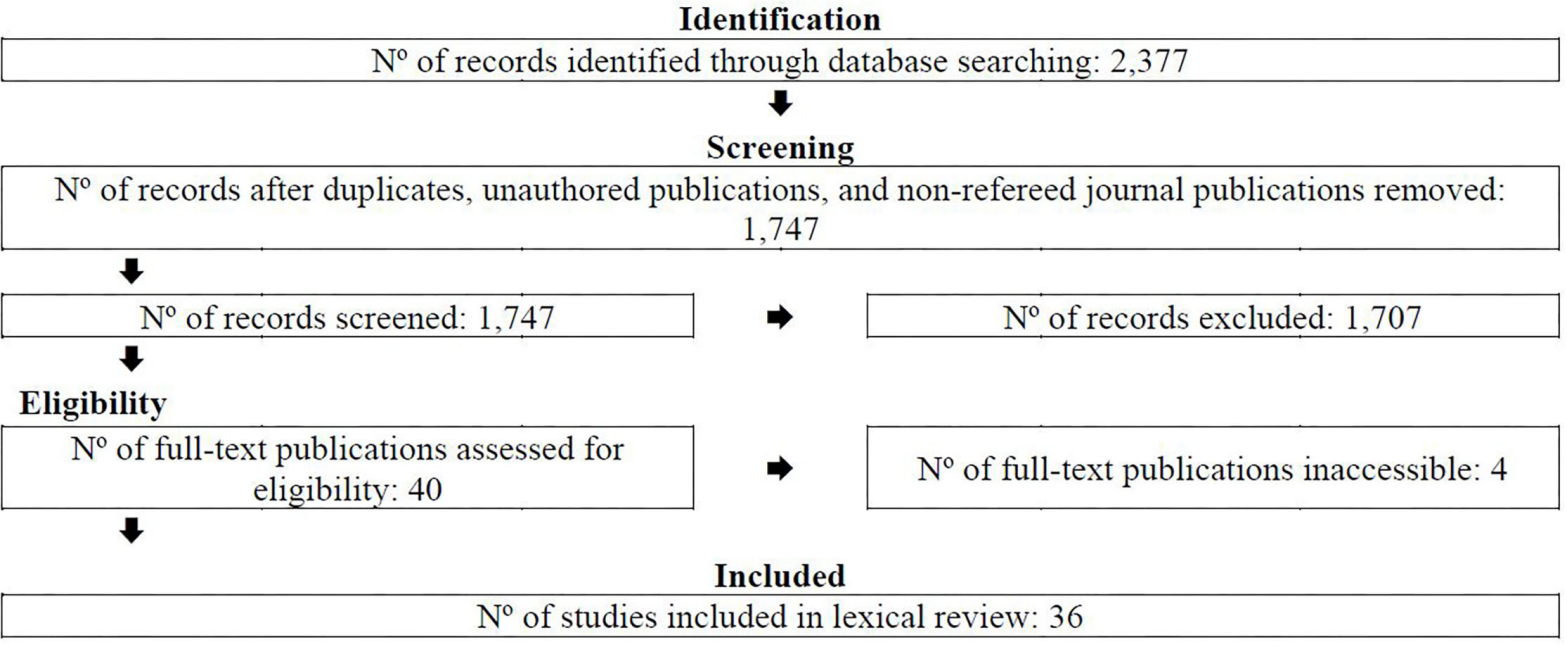
PRISMA flowchart.

Of the 2,377 publications identified via the aforesaid academic databases, 40 met the aforesaid criteria (see Figure 1). To optimize robustness: both authors screened the first 55 publications by reviewing the title and abstract of each identified publication to determine whether it met the aforesaid criteria; discussed their selections; and reconciled differences. Following this, each author screened half of the remaining publications by reviewing the title and abstract of each identified publication to determine whether it met the aforesaid criteria (*n* = 1,692) and conferred about those that warranted discussion. Of the 40 publications deemed to be eligible, 4 were inaccessible and were omitted from the analysis—thus, 36 publications were included in this review. The results section from each publication was then sourced and prepared for a lexical analysis—this involved copying and pasting the text (excluding tables and figures) into a single Word file. Focusing solely on the results section of each publication helped to ensure the lexical analysis was not diluted by potentially redundant text (e.g., a review of extant literature, a description of the methods used, a discussion of implications for others and methodological limitations).

Of the 36 publications, most were published in 2021 (61.1%), rather than 2020 (38.9%), and most presented a study conducted on one continent (86.1%)—chiefly, North America (47.2%). Other continents represented included: Europe (19.4%); Asia (13.9%); Oceania (2.8%); and Africa (2.8%). Several publications reported on a study that was conducted in more than one nation (13.9%). The publications were published in journals pertaining to several different disciplines, including: management (41.7%); healthcare (38.9%); psychology (16.7%); and, to a lesser extent, education (2.8%).

To optimize the likelihood of a systematic approach (9), the lexical analysis was aided by Leximancer—data-mining software that uses Bayesian reasoning to detect key concepts and reveal their relationships (10). Using algorithms, Leximancer identifies frequently occurring and co-occurring words and amalgamates these to form and visually map concepts that reflect themes within the text (11). The maps convey three types of information—“the main concepts in the text and their relative importance; the strengths of links between concepts (how often they co-occur); and similarities in contexts where links occur” (12). Concepts represent “collections of words that generally travel together throughout the text” (13). The components of these concepts are ordered within a thesaurus, comprised of relevant words and weightings to indicate relative importance. Within the map, connections between concepts that are most probable are represented by a spanning tree of gray lines or branches. Clusters of concepts within a map—known as themes—suggest contextual similarity (14). Themes are color-coded to signify those that are (not) important, whereby the “most important theme appears in red, and the next hottest in orange, and so on according to the color wheel” (13).

Leximancer was used in three steps. First, after uploading the Word file into Leximancer, the “discovery” mode was used to, “see what concepts were automatically generated by Leximancer without intervention” (15). Second, Leximancer was used to examine the comparative importance of the concepts, as denoted by relevance percentage. A relevance percentage represents “the percentage frequency of text segments which are coded with that concept, relative to the frequency of the most frequent concept in the list… This measure is an indicator of the relative strength of a concept's frequency of occurrence” (16). Third, the branches that connected concepts germane to this study—namely, *leadership* and *COVID*—were examined.

## Results

**Figure 2:**
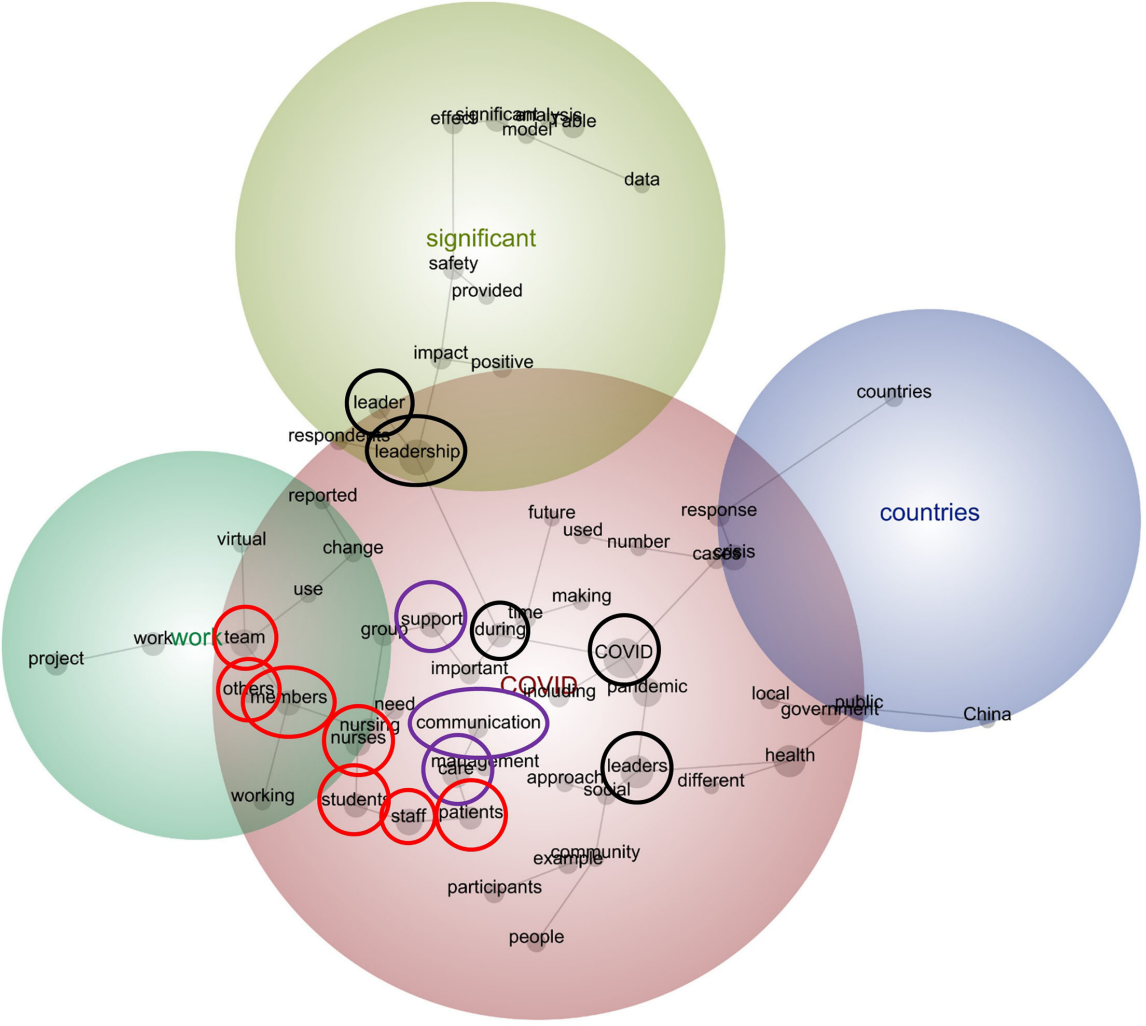
Concept Map (visible concepts: 100%; theme size: 70%).

The concept map at 70% theme visibility and the accompanying thematic summary reveal four themes—namely: *COVID, significant, work*, and *countries* (see Figure 2). These highlight the key clusters of concepts represented within the text. Theme position illustrates the relationships between the themes. Consider the prominence and centrality of *COVID*, which appears in red and overlaps with the remaining three themes. Given that all the publications focused on COVID-19, the prominence of this theme is unsurprising. Its overlap with the remaining themes suggests that, when the publications referred to *COVID* (and the concepts therein), they were inclined to refer to *significant, work*, and *countries* (and the concepts therein):

The command center began a daily outreach via email for up-to-date information to all employees on system-wide **COVID**-19 efforts. Nursing leaders augmented their **work** schedules to increase visibility and support [(17); emphasis added].

It is her ability to communicate purpose to the people of her country in a clear and frequent manner. This can be seen by citizens of New Zealand, such as Christine Nam who said, “Most New Zealanders can verbalize the government's response to **Covid**-19, while the same can't be said for other **countries** because the response has been muddled and indecisive” (Taylor 2020, p. 2) [(18); emphasis added].

While the centrality of the theme, *COVID*, is noteworthy, so too is the distance between the themes, *work* and *countries*. This suggests that when the publications referred to *work* and the concepts therein, like *project*, they were disinclined to refer to *countries* and the concepts therein, like *China*, and vice versa:

The interactions between **work** conditions and communality significantly predicted competence, such that high ratings of communality led to higher competence evaluations for the **work** from home group than the other two groups (see Supplementary Figure 1). However, the interactions between **work** conditions and agency did not predict competence [(19); emphasis added].

In 2017, Forbes reported that China now owns international port holdings in Greece, Myanmar, Israel, Djibouti, Morocco, Spain, Italy, Belgium, Cote d'Ivoire, Egypt, and about a dozen other countries 66. In 2018, China took control of Kenya's largest port after that nation defaulted on its unpaid Chinese loans [(20); emphasis added].

Given the focus of this study, three concepts warrant closer consideration—namely, *leader, leadership*, and *leaders*. Although *leader* and *leadership* are in close proximity to each other, *leaders* is not. This suggests that, while references to *leader* were likely to travel with references to *leadership* (and vice versa), references to *leaders* were less likely to travel with either of these concepts:

While an effective **leader** may not always be an effective manager, the group members agreed that an effective manager should always be able to display effective **leadership** skills [(21); emphasis added].

Frontline administrative **leaders** spearheaded the charge against the COVID-19 with vigor but soon lost tempo due to unfavorable circumstances and preferred to remain in the shadows. Central administrative **leaders** are widely believed to be calling the shots, but they, too, remain largely out of sight [(22); emphasis added].

Furthermore, relative to the concepts, *leader* and *leadership*, the concept, *leaders*, is in closer proximity to that of, *COVID*. As such, discourse pertaining to the pandemic was likely to travel with discourse pertaining to *leaders*, plural, rather than that pertaining to a single leader or leadership:

Nurse **leaders** face a tough road ahead as health care providers grapple with a pandemic. While non-health care workers begin to seek a new normal, the script has not changed for nurses who go to work every day to treat and care for **COVID**-19 patients [(23); emphasis added].

And one of my jobs as a **leader** is to give them their heads, it's a matter of identifying where the strengths are and evolving them. (George) I was really impressed, surprised, overwhelmed by the leadership of our senior leadership team [(24); emphasis added].

Given the important relationship between leadership and followership (25–27), it is curious to note that the concepts, *leader, leadership*, and *leaders* are not closely coupled with the concepts, *team, members*, or *others*. They are not in close proximity, nor are they directly connected. Similarly, *leader, leadership*, and *leaders* are not closely coupled with *nurses, students, staff*, or *patients*. Collectively, these findings suggest that discourse pertaining to leadership did not typically travel with discourse pertaining to these cohorts:

They are made of **team members** who are perseverant and highly motivated. Koser et al. also find that, with these teams, performance is not enhanced by the equipment [(28); emphasis added].

However, many **nurses** reacted positively to this new reality: they strengthened their knowledge base and devised new solutions. Nevertheless, the pandemic has left deep marks in the professional lives of many **nurses** [(29); emphasis added].

Also curious is the position of the concepts, *support, communication*, and *care*. Although effective leaders are touted for their supportive, communicative, and/or caring style, particularly during times of change and uncertainty (30–33), these concepts are not closely coupled with *leader, leadership*, and *leaders*. As such, discourse from the publication results that pertained to leadership did not travel closely with discourse pertaining to support, communication, or care (and vice versa):

Previous pandemics have demonstrated the essential role that crisis **communication** plays in building trust and solidifying the perceived legitimacy of public leaders (Siegrist and Zingg, 2014). In Chile, effective **communication** has been an issue during the pandemic [(34); emphasis added].

For example, if the primary problem had been defined as hospitalizations, and deaths as the consequences, then we might have limited our countermeasures to increasing hospital bed and intensive **care** unit surge capacity as the primary strategy to save lives. In contrast, with a public health prevention mindset, we defined the problem as uncontrolled community transmission of SARS-CoV-2, with the consequences being the number of cases, hospitalizations, and deaths [(35); emphasis added].

Of all the word-like concepts—that is, the concepts that do not denote proper nouns, like *COVID, Table*, and *China*, all of which commence with a capital letter—*leadership* and *leaders* have the greatest relevancy score (see Table 1). Specifically, the concepts, *leadership* and *leaders*, are both 78% relevant to that of *COVID*, which is the most salient (100%):

**Table 1:** Top four ranked concepts.

**Concept**	**Count**	**Relevance (%)**
Name-like concept
COVID	203	100
Word-like concepts
Leadership	159	78
Leaders	159	78
Health	147	72

It appears, therefore, that Germany under Merkel's **leadership** will continue to consider **COVID**-19 to be a serious threat for the foreseeable future [(36); emphasis added].

As noted in the method section, 84% of our participants are **leaders** at institutions that had crisis management plans. Yet, our leaders agreed that these plans were not as helpful as they could be for **COVID**-19 [(37); emphasis added].

The connection between the concepts, *leadership* and *COVID*, is indirect, with the concept, *during*, serving as a nexus between the two, as indicated by the branches. This demonstrates a pathway within the discourse, whereby it was through the global crisis that leadership was demonstrated, experienced, or longed for:

I learned that I needed to LISTEN to my frontline and provide them with the support and trust **during** these difficult times (NE03). Our success in dealing with **COVID**-19 resulted from the flexibility of the nursing leadership in being leaders and being followers [(38); emphasis added].

Collectively, these findings suggest that, although all the publications met the inclusion criteria, discourse pertaining to leadership was not coupled with indications typically associated with leadership. This helicopter view of the publication results suggests that references to leadership in the context of COVID-19 did not travel with references to collaboration with or serving others—nor did they travel with discourse on support, communication, and care.

## Discussion

To manage pandemics, leadership can enable health services to weather the storm. Yet there is limited clarity on how leadership manifested and was discussed in the literature during COVID-19. This can have considerable public health implications given the importance of leadership in the health sector (3–5).

To address this missed opportunity, a lexical analysis was conducted of the results section of relevant publications, identified via a systematic review of nine academic databases. From this, two key findings were revealed. First, among the publications included in this study, *leadership* discourse was often associated with a single *leader*, rather than multiple *leaders*—this is despite the demonstrated relationship between *leaders* and *COVID*, as per the concept map. This reinforces the way in which leadership is often attributed to an individual, rather than to a team of leaders (39).

Second, and related to the previous finding, discourse pertaining to leadership was not closely connected with that pertaining to others. The ways in which leaders and leadership were described were somewhat disconnected from other stakeholders, including colleagues and patients, and relationships with these stakeholders. Consider the separation between the concepts relevant to leaders(ship) and those relevant to particular cohorts—similarly, consider the distance between the concepts relevant to leaders(ship) and the concepts, *support, communication*, and *care.*

Collectively, these findings potentially signal a problem with the ways in which leadership during a pandemic is conceived. Specially, the emerging discourse on COVID-19 appears to place an incredible onus on sole individuals who are unlikely to meet the varied expectations of themselves and others. This can unhelpfully fortify the “cult of leadership” (40).

Progressive understandings of leadership recognize the concept as relational and one of many ways to organize, akin to a “Swiss army knife” (41, 42). For instance, Alvesson and Blom (43) noted that a myopic view of leadership does little to advance its scholarship and practice:

In contrast to many other popular texts on how to “lead” an organization, our suggestion is to move away from a one-sided focus on the manager (as a potential leader) knowing best and viewing leadership as the ultimate key driver, making all the key decisions, including if and how to delegate. Wise forms of organizing need to involve also the non-managers… we emphasize the importance of initiatives from and dialogue with the subordinates… to define and agree upon the appropriate balance between… different modes [of organizing].

Furthermore, these authors argued that continued references to leadership can unhelpfully reinforce an unsophisticated assumption that it is the panacea for organizational woes:

We also, in contrast to most writings on leadership, deliberately use alternative vocabulary to leadership… to address various options… this helps managers and others break away from being trapped in narrow-minded, leadership-infused language and thinking. We strongly warn against the over-use of the term “leadership”… If we look at virtually all leadership and management literatures and listen to the large majority of managers and management educators there is a strong and often naïve belief… that “leaders rule and lead followers.” We need to support alternative vocabularies and mind-sets… Our suggestion [is] to see leadership as just one option and to emphasize both a range of alternatives, and the need to include subordinates in the active work of finding a good combination of alternatives… leadership recipes are attractive and seductive, but [are] seldom… helpful… We have studied many managers creating problems for themselves through a naïve and uncritical belief in seductive leadership ideals (43).

Given the findings from the lexical analysis, the emerging literature on leadership during COVID-19 would benefit from more varied vocabularies and conceptions that reflect the “messiness” of leadership (44). Without this, researchers risk the prospect of promulgating unhelpful scholarship.

Despite the value of the findings presented in this article, four methodological limitations warrant mention. First, the search strategy is unlikely to have identified all relevant articles, given the many potential ways to refer to the key terms (i.e., leadership, pandemic). Second, given the sole use of a lexical analysis, a thematic or critical analysis of the publications is likely to yield different findings. Third, given the study period, the lexical analysis was unlikely to include studies that serve to identify the longer-term effects associated with particular leadership approaches. And fourth, the geographical scope of the publications represented in this study directed attention to better-resourced nations. There is therefore no assumption that the findings have relevance to all nations or continents.

Notwithstanding the aforesaid limitations, a key strength of this study is the use of a lexical analysis to ascertain patterns in academic discourse on leadership during COVID-19. For three key reasons, using Leximancer can be particularly useful during a global pandemic. First, it can simultaneously make sense of “voluminous and disparate bodies of texts” (45)—this benefit is noteworthy, given the exponential growth of the myriad forms and sources of information pertaining to COVID-19, some of which was conflicting. Second, by providing a helicopter view of the discourse, Leximancer can elucidate patterns in how language is used (46)—this can serve to compare different forms and sources of discourse, as well as gauge changes overtime in perception, sentiment, tone, and content. For instance, there is opportunity to test public perceptions and the effects of policy changes using, for instance, a large corpus of media reports, social media, and public health reports. Third, because of the algorithms Leximancer uses, its analyses are less researcher-driven, relative to other approaches, like thematic analysis (47)—this offers a more objective interpretation, reducing the introduction of bias based on assumptions. Given these affordances, lexical analyses using Leximancer can inform research and policymaking, particularly during precarious periods, like a global pandemic.

The findings from this article have clear implications for scholars. Beyond the oft-cited call for more research, what is particularly needed is research that is empirical. This is because, of the 1,707 publications that were excluded from this study, many were: commentaries; conceptual and rhetorical analyses of the performance of political leaders; personal accounts of COVID-19 experiences; or reflections on the leadership of those on the frontline (29, 48–53). This suggests there is considerable opportunity for empirical research, particularly that which will help to clarify different approaches to lead teams and organizations during a pandemic. Additionally, given Alvesson and Blom's (43) advice, research is needed that provocatively draws on diverse vocabularies and conceptions of managing and leading. Rather than continue to situate leadership on select individuals, the time is ripe to problematize, critique, and advance the scholarship and practice of leadership (54, 55).

## Author Contributions

AD conceived the study design, deployed the search strategy, and developed the Sections titled, Methods and Results. AD and AM designed the study as well as developed and tested the search strategy, reviewed the identified publications, identified those that met the inclusion criteria, as well as extracted and analyzed content from the relevant publications. AM developed the Sections titled, Introduction and Discussion. All authors have agreed to be personally accountable for their contributions and ensure that questions related to the accuracy or integrity of any part of the work, even ones they were not personally involved, are appropriately investigated, resolved, and the resolution documented in the literature, and reviewed and approved the final manuscript.

## Conflict of Interest

The authors declare that the research was conducted in the absence of any commercial or financial relationships that could be construed as a potential conflict of interest.

## Publisher's Note

All claims expressed in this article are solely those of the authors and do not necessarily represent those of their affiliated organizations, or those of the publisher, the editors and the reviewers. Any product that may be evaluated in this article, or claim that may be made by its manufacturer, is not guaranteed or endorsed by the publisher.
